# Increased Amino Acid Absorption Mediated by *Lacticaseibacillus rhamnosus* IDCC 3201 in High-Protein Diet-Fed Mice

**DOI:** 10.4014/jmb.2212.12020

**Published:** 2023-01-28

**Authors:** Hayoung Kim, Jungyeon Kim, Minjee Lee, Hyeon Ji Jeon, Jin Seok Moon, Young Hoon Jung, Jungwoo Yang

**Affiliations:** 1 Ildong Bioscience, Pyeongtaek 17957, Republic of Korea; 2 Carl R. Woese Institute for Genomic Biology, the University of Illinois at Urbana-Champaign, Urbana, IL 61801, USA; 3 School of Food Science and Biotechnology, Kyungpook National University, Daegu 41566, Republic of Korea; 4 Ildong Pharmaceutical, Hwaseong 18849, Republic of Korea

**Keywords:** Probiotics, proteolysis, milk protein, *Lacticaseibacillus rhamnosus*, high-protein diet

## Abstract

The use of dietary protein products has increased with interests in health promotion, and demand for sports supplements. Among various protein sources, milk protein is one of the most widely employed, given its economic and nutritional advantages. However, recent studies have revealed that milk protein undergoes fecal excretion without complete hydrolysis in the intestines. To increase protein digestibility, heating and drying were implemented; however, these methods reduce protein quality by causing denaturation, aggregation, and chemical modification of amino acids. In the present study, we observed that *Lacticaseibacillus rhamnosus* IDCC 3201 actively secretes proteases that hydrolyze milk proteins. Furthermore, we showed that co-administration of milk proteins and *L. rhamnosus* IDCC 3201 increased the digestibility and plasma concentrations of amino acids in a high-protein diet mouse model. Thus, food supplementation of *L. rhamnosus* IDCC 3201 can be an alternative strategy to increase the digestibility of proteins.

## Introduction

The protein market is rapidly expanding owing to a constantly increasing interest in health promotion, and rising demand for sports supplements [1]. Among them, milk proteins exhibit several economic and nutritional advantages when compared with meat or vegetable proteins [2]. Meat proteins are produced by processing animals raised over a prolonged period, whereas milk proteins can be supplied continuously from livestock [3]. Milk proteins are more nutritious, given their greater digestibility and higher essential amino acid contents than vegetable proteins [4]. In addition, the production of casein or whey proteins is more economically feasible, as they can be produced from by-products while processing cow or goat milk [5]. Based on these advantages, the whey protein market is expected to expand from $11.0 billion in 2022 to $18.12 billion in 2029 [6].

Dietary proteins play important roles in various physiological processes in humans. However, these proteins need to undergo hydrolysis into small peptides or amino acids by proteases in the gastrointestinal tract (i.e., stomach and intestines) prior to absorption into enterocytes [7]. Subsequently, amino acids are primarily utilized in protein synthesis in the tissues, and synthesized proteins regulate critical biochemical functions, including enzyme reaction, synthesis of low-molecular-weight metabolites [8], energy supply [9], and structural functions [10]. Conversely, protein deficiency can typically cause minor (*e.g.*, fatigue and weakness) to severe symptoms (*e.g.*, liver failure and porous bones).

Although various processing methods such as heating and drying, have been implemented to increase the digestibility of proteins, these methods are known to affect protein quality by causing denaturation, aggregation, and chemical modification of amino acids [11, 12]. Recently, the relationship between probiotics and dietary proteins has been explored, considering protein hydrolysis and subsequent amino acid adsorption. Probiotics provide free amino acids to the host by releasing proteases and peptidases in the intestine [13]. For example, probiotic supplements with a milk protein concentrate diet can enhance the absorption of amino acids, such as arginine, isoleucine, serine, and methionine [14]. In addition, probiotic supplements in a protein diet can reduce toxic metabolites that originate from harmful protein fermentation [15].

In the present study, we examined the effect of probiotic supplements on amino acid absorption in mice receiving a high-protein diet. We first screened the probiotic strain exhibiting the highest proteolytic activity among three candidates using the spot assay in a protein-rich medium. Next, we determined whether co-administering a selected probiotic strain with milk proteins to mice could increase digestibility and thereby enhance the plasma concentration of amino acids. Based on the findings of the present study, consuming probiotics with dietary proteins can be recommended for amino acids absorption in the host.

## Materials and Methods 

### Bacterial Strains and Culture Conditions

The probiotic strains, *Lacticaseibacillus rhamnosus* IDCC 3201 (ATCC BAA-2836), *Streptococcus thermophilus* IDCC 2201 (ATCC BAA-3150), and *Enterococcus faecium* IDCC 2102 (ATCC BAA-3146) were procured from Ildong Bioscience (Korea). Bacterial strains were anaerobically cultured in 14 ml of de Man Rogosa and Sharpe (MRS; BD Difco, USA) medium at 37°C for 24 h in a static incubator.

### Screening of Probiotic Strain with the Highest Proteolytic Activity

To evaluate the proteolytic activities of probiotic strains, a basal medium containing 0.5% (w/v) tryptone, 0.25%yeast extract, 0.1% glucose, 2.5% skim milk powder, and 1.5% agar was used. Then, 10 μl of each probiotic strain culture (10^8^ CFU /ml) was spotted on the agar plate. After incubating the plates at 37°C for 24 h, the length of the clear zone surrounding the colonies was measured using ImageJ software (version 1.8.0, NIH, USA).

### Genome-Wide Identification of Genes Encoding Proteolytic Enzymes

Putative genes for proteolytic enzymes from genome of *L. rhamnosus* IDCC 3201 [16] were predicted using Prokka v1.14.5 [17] and functional annotation was performed using COG (clusters of orthologous groups; NCBI). Additionally, amino acid sequences were submitted to InterProScan v5.30-69.0 and PSI-BLAST v2.4.0 with EggNOG Database v4.5 for genome scale protein function classification [18-20]. Furthermore, to compare the proteolytic genes of other *L. rhamnosus* strains, complete genome sequences of *L. rhamnosus* NCTC 13764T (LR134331.1), *L. rhamnosus* ATCC 53103 (CP031290.1), and *L. rhamnosus* IDCC3201 (CP045531.1) were obtained from the NCBI microbial genome database (https://www.ncbi.nlm.nih.gov/genome).

### Quantification of Compositional Amino Acids in Milk Protein Mixture

For the quantification of amino acids, ninhydrin method was exploited [21]. We employed ion-exchange chromatography to quantify the amino acids in a milk protein mixture comprising whey protein, skimmed goat milk protein, and colostrum protein. The milk protein mixture (0.2 g) was mixed with 10 ml of 6 N HCl in a reactor. After the nitrogen gas injection, the mixture was treated at 110°C for 24 h. The decomposed mixture was concentrated using a vacuum concentrator, adjusted to 50 ml with 0.2 M sodium citrate buffer, and filtered through a 0.45-μm nylon syringe filter. For quantifying 15 amino acids (alanine, arginine, aspartate, glutamate, glycine, histamine, isoleucine, leucine, lysine, phenylalanine, proline, serine, threonine, tyrosine, and valine) except for tryptophan, 20 μl of the filtered sample was injected into Hitachi L-8900 amino acid analyzer with an ultraviolet (UV) detector (Hitachi High-Technologies Corporation, Japan) and an ion-exchange column (#2622PH column 4.6×60 mm). Buffer set for protein hydrolysate, PH-SET KANTO (Hitachi High-Technologies Corporation) which is used to elute amino acids in the ninhydrin method, was used as a mobile phase. Flow rates of the buffer set and ninhydrin buffer were 0.35 and 0.40 ml/min, respectively, and protein peaks were detected at 440 and 570 nm. To quantify tryptophan, high-performance liquid chromatography (Agilent 1260, Agilent Technologies, USA) was performed using a CAPCELL C18 (250 × 4.6 mm, Osaka Soda, Japan) column. As mobile phases, 8.5 mM sodium acetate and methanol (95:5, v/v) were used at a flow rate of 1.0 ml/min. The peak of tryptophan was detected at 280 nm using a UV detector. To quantify methionine and cysteine, the sample was pulverized using a sample grinder and passed through a sieve with a particle size of 500 μm. The sieved sample (2 g) was mixed with acetonitrile and water solution (1:9, v/v; 50 ml), followed by sonication for 20 min at 45°C. The sonicated sample was centrifuged at 3,000 ×*g* for 20 min. Subsequently, 5 ml of the supernatant was transferred to a 15 ml test tube, and 100 μl of 50% (w/v) KOH was added and stirred for 10 s. After adding 100 μl of 85% (w/v) phosphoric acid to the sample, the supernatant was filtered through a 0.45-μm nylon syringe filter. Next, 20 μl of the sample was injected into a Hitachi L-8900 amino acid analyzer with a UV detector (Hitachi High-Technologies Corporation). An NH_2_ column (reversed-phase, 150 mm × 4 mm, Altman Analytik, Germany) was used, and 0.01 M H_3_PO_4_ (pH 3.2) and acetonitrile solution (isocratic mode; ratio 77:23, v/v) were employed as the mobile phase at a constant flow rate of 1 ml/min. Finally, the peaks of methionine and cysteine were detected at 214 nm using a UV detector.

### Mouse Experiments

Six-week-old male C57BL/6J mice were purchased from Central Lab Animal Inc. (Korea) and maintained in a controlled atmosphere with a 12:12 h light and dark cycle. The mice were fed either a normal diet (ND; 2018S Teklad Global 18% Protein Rodent Diet; Envigo, USA) or a high-protein diet (a mixture of ND and milk protein blends; 6:4 w/w) for eight weeks. Mice (*n* = 24) were randomly divided into four groups: 1) ND; 2) High-protein diet (HPD); 3) HPD + *L. rhamnosus* IDCC 3201 (HPD + LLrh; 10^7^ CFU/day); 4) HPD + *L. rhamnosus* IDCC 3201 (HPD + HLrh; 10^8^ CFU/day). ND or HPD were supplied *ad libitum*, and *L. rhamnosus* IDCC 3201 was orally administered to mice daily. Animal studies were approved by the Institutional Animal Care and Use Committee of the Chungbook National University (approval number: CBNUA-1687-22-02). The body weight, and water and food consumption were measured weekly. After eight weeks, mice were subjected to a 6 h fasting period and anesthetized with diethyl ether. Blood samples were collected from the abdominal veins and centrifuged at 600 ×*g* for 20 min. The serum was collected and stored at −70°C until further use. To analyze free amino acids in serum, we employed a Hitachi L-8900 amino acid analyzer with a UV detector (Hitachi High-Technologies Corporation, Japan) and an ion-exchange column (#2622PH column 4.6 × 60 mm).

### Statistical Analyses

Results are expressed as mean ± standard deviation (SD). For statistical data comparisons, analysis of variance (ANOVA) analysis followed by Duncan test and Student’s *t*-test were performed using STATISTICA version 7 (TIBCO Software Institute, USA).

## Results 

### Screening of *Lacticaseibacillus rhamnosus* IDCC 3201 with Higher Proteolytic Activity

**Fig. 1:**
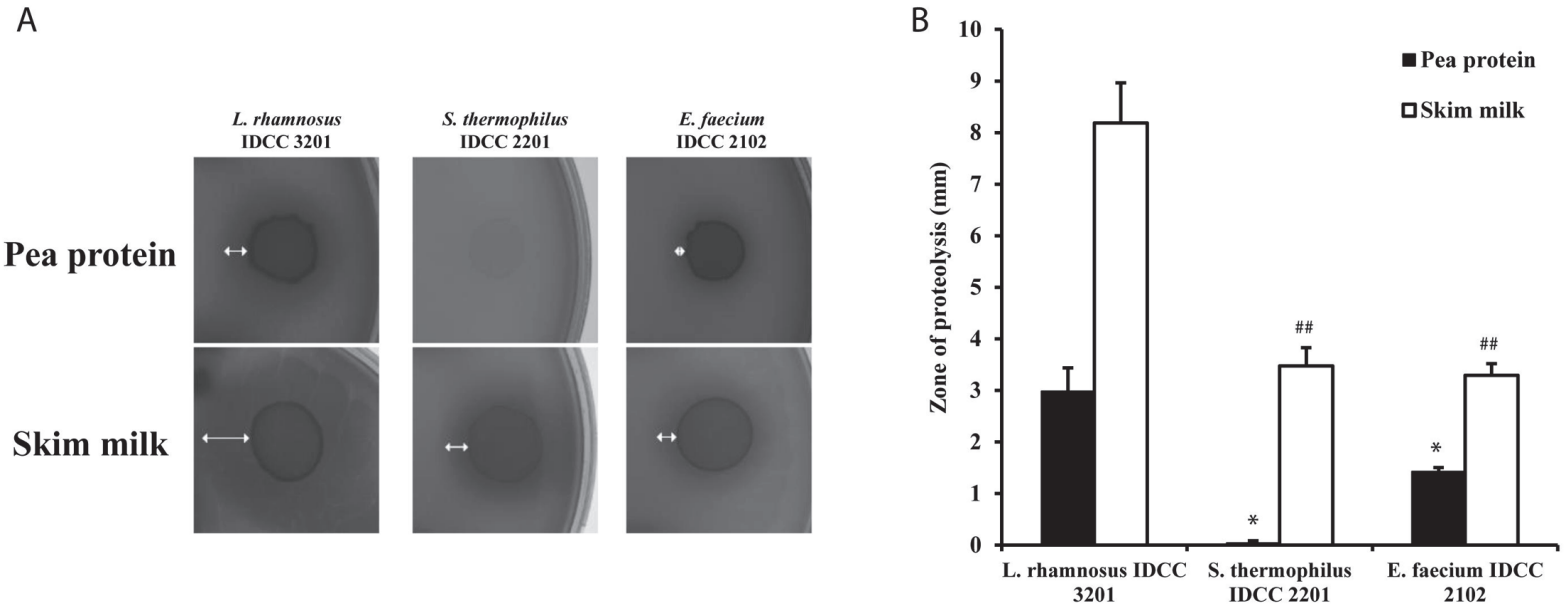
Proteolytic activity of *Lacticaseibacillus rhamnosus* IDCC 3201, *Streptococcus thermophilus* IDCC 2201, and *Enterococcus faecium* IDCC 2102 in pea protein and skim milk agar medium. (**A**) One representative clear zone data set for three independent experiments. (**B**) Each value is an average of triplicate experiments with data presented as mean ± standard deviation (SD). (*, *p* < 0.05; **, *p* < 0.01 vs. *L. rhamnosus* IDCC 3201 in pea protein and ##, *p* < 0.01 vs. *L. rhamnosus* IDCC 3201 in skim milk).

To select an optimal probiotic strain capable of improving milk protein digestion, the proteolytic activities of *L. rhamnosus* IDCC 3201, *S. thermophilus* IDCC 2201, and *E. faecium* IDCC 2102 were evaluated. Each probiotic strain was spotted on a basal agar medium containing skim milk and incubated for 24 h to measure the size of the halo produced by proteolysis. In results, *L. rhamnosus* IDCC 3201 produced an 8.18 ± 0.78 mm halo surrounding the colony, whereas *S. thermophiles* IDCC 2201 and *E. faecium* IDCC 2102 produced halos of 3.47 ± 0.35 and 3.29± 0.22 mm, respectively (Fig. 1A). The *L. rhamnosus* IDCC 3201 generated halo via proteolysis of skim milk was 2.4- and 2.5-fold larger than those generated by *S. thermophiles* IDCC 2201 and *E. faecium* IDCC 2102, respectively (Fig. 1B). Considering pea protein, *L. rhamnosus* IDCC 3201 also exhibited the highest proteolytic activity among examined strains (Fig. 1B). In order to investigate the cause of the higher proteolytic activity of *L. rhamnosus* IDCC 3201 beyond the other probiotic strains, the genes of protease, peptidase, and proteinase of representative *L. rhamnosus* strains, *L. rhamnosus* NCTC 13764, *L. rhamnosus* ATCC 53103, and *L. rhamnosus* IDCC 3201 were investigated (Table 1). As a result, the genome of *L. rhamnosus* NCTC 13764 contained 13 proteases, 38 peptidases, and 2 proteinases, the genome of *L. rhamnosus* ATCC 53103 contained 12 proteases, 40 peptidases, and 2 proteinases, and the genome of *L. rhamnosus* IDCC 3201 contained 13 proteases, 40 peptidases, and 2 proteinases. In particular, *L. rhamnosus* IDCC 3201 had serine protease/ABC transporter B family protein *tagC*, probable endopeptidase *YddH*, and Beta-Ala-His dipeptidase, which are absent in other strains.

### *L. rhamnosus* IDCC 3201 Increased Amino Acid Absorption in HPD-Fed Mice

To determine whether the co-administration of selected probiotic and milk proteins could improve protein digestibility in HPD mice, we prepared a milk protein mixture by mixing whey protein, skimmed goat milk protein, and colostrum protein. The amino acid composition of the protein mixture were as follows: tryptophan 1.3% (w/w), glycine 1.7%, histamine 1.8%, cysteine 3.3%, tyrosine 2.3%, methionine 2.3%, arginine 2.4%, phenylalanine 3.2%, serine 4.7%, alanine 5.1%, valine 5.5%, isoleucine 5.9%, proline 6.6%, threonine 6.6%, lysine 9.0%, aspartate 10.3%, leucine 10.5%, and glutamate 17.6% (Table 2).

**Fig. 3:**
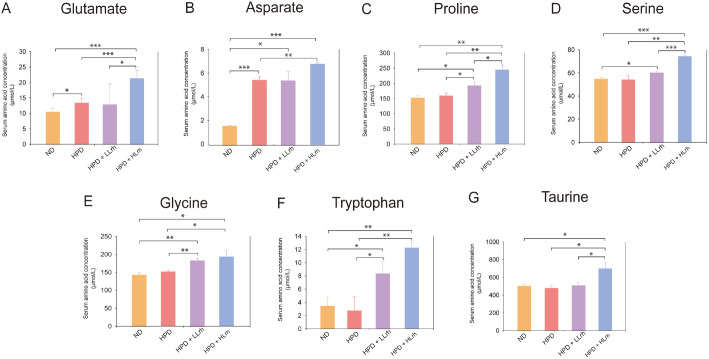
Seven amino acids as serum biomarkers for amino acid absorption in the high protein diet mouse model treated with *Lacticaseibacillus rhamnosus* IDCC 3201. The concentration of 7 amino acids in serum after HPD diet treatment or *L. rhamnosus* IDCC 3201 administration. ND: Normal diet group; HPD: High-protein diet group; HPD + *L. rhamnosus* IDCC 3201 (HPD + LLrh; 5×10^7^ CFU/day); HPD + *L. rhamnosus* IDCC 3201 (HPD + HLrh; 5×10^8^ CFU/day). Data values are expressed as mean ± standard deviation (SD). (*, *p* < 0.05; **, *p* < 0.01; ***, *p* < 0.001).

During the eight-week experimental period, we observed no significant difference in the food efficiency ratio (body weight gain (g)/food intake (g) × 100) between examined groups. To determine whether the co-administration of *L. rhamnosus* IDCC 3201 and the milk protein mixture would enhance protein digestibility in HPD diet-fed mice, plasma concentrations of amino acids were quantified using an amino acid analyzer. Firstly, the HPD group showed significantly increased aspartate and glutamate absorption than the ND group (Fig. 3). Next, co-administration of *L. rhamnosus* IDCC 3201 and milk protein mixture resulted in significant increased serum concentration of glycine, proline, and tryptophan in both HPD + LLrh and HPD + HLrh groups by up to 27.47, 54.01, and 64.92%, respectively, compared to those in the HPD group. Meanwhile, aspartate, glutamate, serine and taurine concentrations were only elevated in HPD + HLrh group by 23.59, 60.45, 36.95, and 47.29%, respectively, compared to those in the HPD group (Table 3).

## Discussion

Based on the notion that intestinal microbes degrade dietary proteins and in turn this promotes the intestinal amino acid absorption in the host [7], this study investigated whether co-administration of probiotics and proteins would increase serum amino acid concentration in high-protein diet-fed (HPD) mice. In this study, *Lacticaseibacillus rhamnosus* IDCC 3201 was selected as a potential probiotic bacterium that can increase amino acid absorption in the host (Fig. 1). Genome analysis indicates that this strain has specific proteases and peptidases. Especially, serine proteases are thought to be main enzymes of protein degradation, because their signaling molecules for extracellular secretion are a member of triggering and cleaving family of proteinase-activated receptors (Table 1) [22]. Furthermore, the proteolytic enzymes such as PepO, IspA, and RseP were founded only in *L. rhamnosus* IDCC 3201, compared to other rhamnosus strains. PepO has distinct cleavage site for a_s1_-casein fragment 1-23, and the protease IspA, which is frequently found mainly in the *Bacillus* species, is crucial role in stationary phase, where cell growth being to stop [24, 25]. Lastly, RseP stimulates transcriptional factor of σ^E^ extra-cytoplasmic stress response and in turn, eliminates signal peptides of extracelluar proteins in the secondary processing in cytoplasmic membrane [26]. On the other hands, The PepR and PepX which are proline-specific peptidases are found in only other *L. rhamnosus* strains, while PepXP (prolyl dipeptidyl aminopeptidase) are only found in in *L. rhamnosus* IDCC3201 and these genes are frequently found in *Lactococcus lactis* strains [27, 28].

Milk protein mixture used in this study is mainly composed of glutamate (17.6%, w/w), leucine (10.5%), aspartate (10.3%), and lysine (9.0%) (Table 1), and this protein composition is similar to the previously reported milk protein mixture, mainly composed of leucine, glutamate, aspartate, and proline [29]. In consistent with amino acid composition of the tested protein mixture, HPD-animal experiments showed significantly increased serum glutamate and aspartate, compared to normal diet group (Fig. 3). Furthermore, co-administration of *Lacticaseibacillus rhamnosus* IDCC 3201 and milk protein mixture increased 7 amino acid absorption of in high-protein diet-fed (HPD) mice: aspartate, glutamate, serine, glycine, taurine, proline, and tryptophan. Among the essential amino acids, only tryptophan concentration increased up to 4.6-fold in co-administration group, compared to HPD group, whereas among the branched-chain amino acids, there was no significantly increased amino acid (Table 2).

Participants in clinical trials who ingested 25 g of a milk protein and 10^9^ CFU of *B. coagulans* GBI-30 had increased concentrations of arginine, isoleucine, serine, ornithine, and methionine in their serum, compared to those who ingested only protein [14]. Moreover, ingestion of a 20g of plant protein and 10 billion CFU of probiotics consisting of *L. paracasei* LP-DG (CNCM I-1572) and *L. paracasei* LPC-S01 (DSM 26760) improved serum concentration of methionine, histidine, valine, leucine, and isoleucine [13]. Together with these results, co-ingestion of either (both) probiotic(s) or (and) protein changes of composition of microbiota in the intestine. For example, high protein diet led to an increase in the proportion of *Bifidobacterium* spp. and *Lactobacillus* spp., whereas, ingestion of *Bacillus* spp. increased the population of healthy gut-related bacteria [30].

Amino acids are crucial for growth development, and health of the host, exerting various regulatory functions in cells including protein synthesis, cell signaling, metabolic regulations, and immune function [31]. With regards to immune function, aspartate and glutamate play versatile roles in the metabolism of leucocytes and lymphocytes [32]. Serine is crucial for several cellular functions, including neurotransmission, folate and methionine cycles, and sphingolipid synthesis [33]. Glycine, synthesized from serine, is a biosynthetic intermediate for protein (*e.g.*, collagen) and is an inhibitory neurotransmitter in the central nervous system [34]. Taurine, synthesized from methionine and cysteine metabolism, is essential for various physiological functions, including glycolysis, glycogenesis, osmoregulation, anti-oxidation, and detoxification [35]. Proline mediates key functions in protein function and the regulation of cellular redox homeostasis [36]. Lastly, tryptophan can be converted into 3-idolepropionic acid (IPA), indole-3-aldehyde (I3A), and indole by gastrointestinal microbes: IPA functions as a neuroprotectant; I3A maintains mucosal reactivity; and indole associated with vascular and chronic kidney diseases [37].

Collectively, our finding suggests that the co-administration of *L. rhamnosus* IDCC 3201 and dietary proteins confer health benefits in terms of amino acid absorption through intestinal enterocytes.

## Figures and Tables

**Fig. 2:**
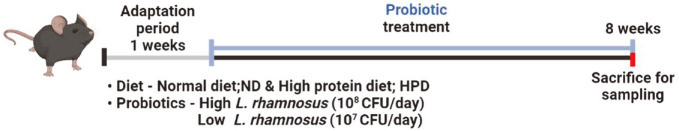
Mouse experiment co-administering a high-protein diet with *Lacticaseibacillus rhamnosus* IDCC 3201.

**Table 1:** The proteolytic genes in various *Lacticaseibacillus rhamnosus* strains.

Enzyme	Gene	*Lacticaseibacillus rhamnosus*		

		NCTC 13764	ATCC 53103	IDCC3201
Rhomboid Protease	*gluP*	5	3	2
Probable inactive metalloprotease YmfF		1	1	1
ATP-dependent Clp protease ATP-binding subunit ClpX		2	1	2
Serine protease/ABC transporter B family protein tagA		0	1	1
Serine protease/ABC transporter B family protein tagC	*ecfA1*	0	0	1
Probable protease eep	*rseP*	1	1	1
ATP-dependent Clp protease ATP-binding subunit ClpA homolog CD4A, chloroplastic		1	1	1
ATP-dependent Clp protease ATP-binding subunit ClpL		1	1	1
Uncharacterized protease YdeA		0	1	1
Probable ATP-dependent Clp protease ATP-binding subunit		1	1	1
ATP-dependent zinc metalloprotease FtsH	*ftsH|hflB*	1	1	1
Peptidoglycan DL-endopeptidase CwlO	*cwlO*	1	1	1
Dipeptidase A	*pepDA|pepDB*	1	1	2
Muramoyltetrapeptide carboxypeptidase	*ldcA*	1	1	1
Pyroglutamyl-peptidase I	*pcp*	2	2	2
Serine-type D-Ala-D-Ala carboxypeptidase	*dacC|dacA|dacD*	6	6	7
Oligoendopeptidase F like protein	*pepF|pepB*	1	2	2
Signal peptidase I	*lepB*	2	2	1
Xaa-Pro aminopeptidase	*pepP*	2	2	2
Putative aminopeptidase YsdC	*sgcX*	1	0	0
Tripeptide aminopeptidase	*pepT*	1	1	1
Membrane alanyl aminopeptidase	*pepN*	1	1	1
Group B oligopeptidase PepB	*pepF|pepB*	1	1	2
Prolyl aminopeptidase	*pip*	3	4	3
Xaa-Pro dipeptidase	*pepQ*	2	2	1
Endopeptidase Clp	*clpP|CLPP*	1	1	1
Aminopeptidase	*ampS|pepS|ampT*	1	1	1
Methionyl aminopeptidase	*map*	1	1	1
C-terminal processing peptidase	*prc|ctpA*	1	1	1
HslU--HslV peptidase	*hslV|clpQ*	1	1	1
Signal peptidase II	*lspA*	1	1	1
Xaa-Pro dipeptidyl-peptidase	*pepX*	1	1	1
Acylaminoacyl-peptidase	*APEH*	1	1	1
Probable dipeptidase B	*pepDA|pepDB*	2	2	1
Putative membrane peptidase YdiL		1	1	1
Peptidase Do	*degP|htrA*	1	1	1
D-Ala-D-Ala dipeptidase	*vanX*	1	1	0
Endopeptidase La	*Ion*	0	1	1
leader peptidase (prepilin peptidase) / N-methyltransferase	*comC*	0	1	0
Probable endopeptidase YddH		0	0	1
Beta-Ala-His dipeptidase		0	0	1
Putative zinc metalloproteinase in scaA 5'region	*pepO*	2	2	2

**Table 2:** The amino acid composition of milk protein mixture used in this study.

Amino acids	Unit (g/100 g)	Percentage (%)
Glutamate	14.15 ± 0.08	17.6
Leucine	8.43 ± 0.08	10.5
Aspartate	8.27 ± 0.02	10.3
Lysine	7.29 ± 0.07	9.0
Proline	5.3 ± 0.01	6.6
Threonine	5.32 ± 0.43	6.6
Isoleucine	4.73 ± 0.37	5.9
Valine	4.45 ± 0.36	5.5
Alanine	4.15 ± 0.02	5.1
Serine	3.79 ± 0.74	4.7
Cysteine	2.69 ± 0.05	3.3
Phenylalanine	2.54 ± 0.13	3.2
Arginine	1.90 ± 0.02	2.4
Methionine	1.85 ± 0.03	2.3
Tyrosine	1.83 ± 0.45	2.3
Histidine	1.43 ± 0.05	1.8
Glycine	1.41 ± 0.01	1.7
Tryptophan	1.08 ± 0.02	1.3
Total essential amino acid	37.10 ± 0.66	46.0
Total non-essential amino acid	43.50 ± 1.15	54.0
Total amino acid	80.60 ± 0.50	100

Each data is presented as mean ± standard deviation (SD).

**Table 3:** Serum amino acid concentration in mice experiments.

Amino acid (μmol/l)	ND^1^	HPD^2^	HPD + LLrh^3^	HPD + HLrh^4^
Glutamate	10.28 ± 1.39	13.21 ± 1.67	12.73 ± 0.31	21.20 ± 0.14***
Leucine	68.86 ± 7.77	64.15 ± 16.99	62.40 ± 1.75	58.80 ± 1.29
Aspartate	1.60 ± 0.09	5.47 ± 0.30	5.42 ± 0.08	6.76 ± 0.09**
Lysine	144.64 ± 18.24	118.09 ± 12.37	117.05 ± 1.76**	118.18 ± 2.35
Proline	150.64 ± 8.56	157.74 ± 9.98	191.31 ± 0.85*	242.14 ± 1.24**
Threonine	75.81 ± 5.90	74.86 ± 12.36	66.60 ± 0.93*	75.41 ± 1.19
Isoleucine	39.01 ± 5.91	36.87 ± 9.34	36.08 ± 0.82	36.86 ± 0.59*
Valine	82.76 ± 40.97	97.59 ± 15.09	94.20 ± 1.68	94.56 ± 1.30
Alanine	188.52 ± 53.10	187.12 ± 19.47	178.27 ± 1.40*	154.30 ± 1.70
Serine	53.91 ± 1.81	53.74 ± 3.98	59.73 ± 0.29	73.60 ± 0.60**
Cysteine	31.58 ± 60.02	5.07 ± 4.74	8.30 ± 0.13*	0.00 ± 0.00
Phenylalanine	37.89 ± 6.16	29.73 ± 6.01	30.87 ± 0.43*	24.25 ± 0.39
Arginine	121.25 ± 10.92	96.97 ± 5.00	95.33 ± 1.22	95.01 ± 1.33
Methionine	25.08 ± 11.77	24.75 ± 2.54	22.63 ± 0.38	24.54 ± 0.48
Tyrosine	53.36 ± 8.62	44.05 ± 2.58	43.03 ± 0.82*	42.16 ± 1.18
Histidine	32.50 ± 10.44	26.34 ± 2.53	28.72 ± 0.21	24.88 ± 0.24
Glycine	141.29 ± 7.21	148.83 ± 5.62	181.20 ± 0.48**	190.99 ± 0.70*
Tryptophan	3.35 ± 1.35	2.63 ± 2.14	8.27 ± 2.14*	12.12 ± 0.31**
Asparagine	0.07 ± 0.01	0.06 ± 0.00	0.06 ± 0.02	0.07 ± 0.01
γ-Aminobutyric acid	0.53 ± 0.24	0.34 ± 0.36	0.64 ± 0.25	0.00 ± 0.00
Glutamine	3329.40 ± 885.59	3430.71 ± 4.83	3495.87 ± 290.69**	2660.22 ± 293.33*
Taurine	499.49 ± 25.77	468.46 ± 32.48	503.47 ± 2.53	690.01 ± 3.57*

Each value is presented as mean ± standard deviation (SD).

^1^Normal diet (ND) group

^2^High-protein diet (HPD) group

^3^High-protein diet (HPD) and orally administered *L. rhamnosus* IDCC 3201 (10^7^ CFU/day; LLrh)

^4^High-protein diet (HPD) and orally administered *L. rhamnosus* IDCC 3201 (10^8^ CFU/day; HLrh)

*Significant at *p* < 0.05; **significant at *p* < 0.01; ***significant at *p* < 0.001 compared with high-protein diet group.
